# Cloning and expression analysis of DnMSI1 gene in orchid species *Dendrobium nobile* Lindl

**DOI:** 10.1080/15592324.2021.2021649

**Published:** 2022-01-10

**Authors:** Baolu Cui, Min Huang, Chongdai Guo, Ruihong Li, Yuqi Wang

**Affiliations:** a School of Environmental Science and Engineering, Guangzhou University, Guangzhou, Guangdong, China; b School of Biological Science and Agriculture, Qiannan Normal University, Duyun, Guizhou, China

**Keywords:** DnMSI1, clone, expression, stress, *Dendrobium nobile* Lindl

## Abstract

WD40 repeat proteins, the homologs of yeast MSI1, are conserved in plants, participating in protein complexes and playing fundamental functions in plant development. Although several MSI1-like proteins have been cloned and characterized in plants, the roles of MSI1-like proteins in the biennial ornamental plant, *Dendrobium nobile* Lindl, are still unclear. Here, we report the cloning of the *DnMSI1* gene from *Dendrobium nobile* Lindl with RACE technology. We found that *DnMSI1* expression was induced by GA_3_ and TDZ but inhibited by ABA, PP333, and drought and salt stress. Furthermore, *DnMSI1* over-expression in *Arabidopsis* resulted in decreased tolerance to NaCl stress. These results suggest that DnMSI1 plays negative regulation roles in regulating salinity-stress resistance in *Dendrobium nobile* Lindl.

## Introduction

MSI1-like proteins of the WD40 family are prevalent in all eukaryotes. They play fundamental roles in plant development, including determining cell fate and regulating cell cycle, signal transduction, transcription, and cytoskeletal organization.^1–3^ MSI1 proteins are subunits of several protein complexes. For instance, Arabidopsis MSI1 (AtMSI1) is a subunit of Chromatin Assembly Complex CAF-1^3,4^ and PRC2 (Polycomb repressive complex 2)-like complexes, which interact with the Retinoblastoma-related protein RBR1^5,6^ and participate in various aspects of chromatin assembly and dynamics.^2^ Reduced expression of MSI1 leads to pleiotropic phenotypes, reflecting the complexity of MSI1 protein functions. AtMSI1is the part of FIS (Fertilization Independent Seed) and CLF (CURLY-LEAF) complex, playing a critical role in seed development. In Arabidopsis, when a mutant allele *msi1* is inherited from the mother, the seeds are aborted regardless of the paternal contribution.^7–9^ Furthermore, heterozygous *Atmsi1* mutants also show a high penetrance of fertilization-independent defects in seed development.

AtMSI1 is also a subunit of the CUL4-DDB1A-MSI1 protein complex, essential for seed development.^7,10^ Reducing *AtMSI1* levels by co-suppression to about 5% of the wild-type levels causes severe defects in vegetative and reproductive development, leading to sterility.^1^ Furthermore, transgenic *AtMSI1* antisense plants resulted in late flowering and increased trichome branching, suggesting that AtMSI1 regulates flowering time and trichome development.^6,11,12^

In addition, Alexandre et al. (2009) revealed a new role of AtMSI1 in regulating drought-stress responses as indicated by increased drought tolerance of the *msi1-cs* mutant plants because of enhanced expression of osmotic stress-responsive genes and accumulation of free proline.^13^ Therefore, identifying stress-inducible and MSI1-regulated genes will help us better understand how chromatin modifications affect plant responses to stress.

Presently, plant MSI1-like genes have been cloned from *Arabidopsis thaliana, Nicotiana tabacum, Hieracium caespitosum, Triticum aestivum, Chlamydomonas reinhardtii, Zea mays, Glycine max, Hieracium pilosella, Oryza Sativa*, etc. Still, there is no report about MSI1 from *Dendrobium nobile*, an important economic orchid in flower markets worldwide with high ornamental and medicinal values. Studies of gene regulation by chromatin modifications in *Dendrobium nobile* may provide guidelines to control their growth status in the future. In this study, we reported the isolation of an *MSI1* gene from *Dendrobium nobile* using a functional genomics approach and the analysis of its expression and interactions with plant hormones and growth regulators.

## Materials and methods

### Plant materials and growth conditions

*D. nobile* plants were grown in moss media in a greenhouse with temperature maintained at 22°C and 18°Cduring day and night. The relative air humidity and light period were 75% and 12:12 (day: night), respectively. For treatment experiments, plants with finished floral bud differentiation were selected and treated with 1 mg/L Gibberellin A3(GA3), 1 mg/L Thidiazuron (TDZ), 1 mg/L Abscisic Acid (ABA), 1 mg/L Paclobutrazol(PP333), drought, 10 μM CdCl_2_, 10 μM NiCl_2_ and 10 mM NaCl, the distilled water as a negative control. After the treatment, plants were transferred to a growth chamber with the same growing conditions. The roots, stems, leaves and buds were collected on 5d for RNA isolation and expression patterns analysis. After different treatments of 0d, 5d, 10d, 15d, 20d, 25d, and 30d, the buds were collected separately and frozen in liquid nitrogen for further analyses. Each treatment contains 6 plants with three biological repeats.

The wild type (Col) *Arabidopsis thaliana* were acquired from the Arabidopsis Biological Resource Center (ABRC) (https://abrc.osu.edu/). Homozygosity of each DnMSI1-overexpression line (AtOE) was confirmed by PCR-based sequencing. Wild-type (Col-0) and AtOE seeds were surface-sterilized and cold stratified in the dark at 4 °C for 3 days before sown on 250 μm polypropylene meshes floating on hydroponic growth solutions supplemented without or with NaCl in Magenta boxes. The hydroponic solution consisted of the following macronutrients in mM: MgCl_2_, 3.0; (NH_4_)_2_SO_4_, 0.25; Ca(NO_3_)_2_, 1.0; KCl, 2.0; CaCl_2_, 2.75; KH_2_PO_4_, 0.18; and the following micronutrients in μM: H_3_BO_3_, 5.0; MnSO_4_, 1.0; CuSO_4_, 0.05; ZnSO_4_, 0.2; Na_2_MoO_4_, 0.02; CoCl_2_, 0.001. Plants were grown in a plant growth chamber with 16/8 h day/night at 22°C.

Relative root growth (RRG%) of Arabidopsis was calculated as the percentage of root growth of individual plants under NaCl treatment over the average root growth under control (−NaCl) condition. In detail, ~30–40 seeds (technical replicates) of the WT or the DnMSI1-overexpressing lines were germinated in a hydroponic solution in a Magenta box supplemented with or without 30–300 mM NaCl. Three biological replicates (Magenta boxes) were conducted for each treatment and line. Primary root lengths of 10 randomly selected 7-d-old seedlings from a biological replicate were measured manually. The means of the primary root length of three biological replicates were calculated for the control of each line. The RRG% of a randomly selected 7-d-old seedling under treatment condition was calculated as the individual primary root length divided by the mean root length of the same line of the control condition. Ten seedlings from each biological replicate were randomly selected for RRG% calculation. The presented RRG% data were the means of three biological replicates.

### RNA isolation and cDNA synthesis

Total RNA was isolated using the TRIzol method.^14^ RNA quality and concentration were determined using ethidium bromide (EB)-stained agarose gel electrophoresis and a UV spectrometer. The first strand of cDNA was synthesized using a Prime Script® RT-PCR Kit (TaKaRa, Dalian, China) with Oligo dT(18) as a primer.

### Isolation of DnMSI1 gene in *D. nobile* Lindl

Total RNAs isolated from frozen *D. nobile* Lindl buds were used as templates for reverse transcription polymerase chain reaction (RT-PCR). The product (the first-strand cDNA) was subjected to PCR amplification by forward primer DnMSI1f1: (5ʹ-CACCTATTGAGTGGTTCTGAC-3ʹ) and reverse primer DnMSI1r1:(5ʹ-ATCTTCTGCATCCTCAGGTG-3ʹ), which were designed with the conserved motifs of *Nicotiana tabacum* MSI1 (multicopy suppressor of IRA1) gene (ABY84675.1). The 3ʹ and 5ʹ end of DnMSI1 was isolated with a 3ʹ and 5ʹ rapid amplification of cDNAs ends (RACE) cDNA Amplification Kit (TaKaRa, Shiga, Japan), respectively. In addition, gene-specific primers of DnMSI1f2 (5ʹ-GTAGCGACAGGTTCAACTGA-3ʹ), and DnMSI1f3 (5ʹ-ATACTTGCATCTTGTTGCC-3ʹ) were used for 3ʹ RACE, while DnMSI1r2 (5ʹ-TTGATCATCACCAACAGAG-3ʹ) and DnMSI1r3 (5ʹ-GCATATTTGAGCATCGTCAG-3ʹ) for 5ʹ RACE. The amplified fragment was recovered from agarose electrophoresis with an Agarose Gel DNA Extraction Kit (RealTims, China) and cloned into the pMD18-T vector (TaKaRa, Dalian, China). The full-length *DnMSI1* cDNAs were obtained by PCR amplification using the forward primer: 5ʹ-ATGGCCAAGGATGAAGATGAC-3ʹand reverse primer: 5ʹ-TTAAGAGGCTTTTGAAGGCTCG-3ʹ.

### Sequence analysis

WD40 protein sequences were downloaded from GeneBank for comparison. Sequence alignment was performed using DNAssist Package version 2 and CLUSTAL.W (http://www-igbmc.u-strasbg.fr/BioInfo/ClustalX) Sequence relatedness was analyzed using CLUSTAL.W and the neighbor-joining method, and the rooted tree was visualized using Treedraw (http://taxonomy.zoology.gla.ac.uk/rod/treeview.htm).

### Quantitative real-time RT-PCR analysis

Quantitative real-time RT-qPCR was carried out using SYBR Premix Ex Taq Kit (TaKaRa, Shiga, Japan). The amplification conditions were 95°C for 30 s, followed by 40 cycles of amplification (95 °C for 5 s, 60°C for 20 s) and plate reading after each cycle. Primer pairs used for quantitative real-time RT-qPCR detection are as follow: forward primer DnMSI1RTf: 5ʹ-TGGAGTCCCAAGAATGAGAC-3ʹ and reverse primer DnMSI1RTr: 5ʹ-ATCTTGCTAGTGTGTCCGCC-3ʹ. In addition, the *UBQ* (*Ubiquitin*) was used for normalization with forward primer (5ʹ-CGCCGTCAACCTCATTCCAT-3ʹ) and reverse primer (5ʹ-GTGAGGTAGCGACCGTGGC-3ʹ). As for detection of *DnMSI1* transcript levels in wild-type (WT) and *DnMSI1* overexpression *Arabidopsis* lines (AtOE lines), the *Arabidopsis tubulin-2* gene was used for calibration with forward primer (5ʹ-ACCACTTTCGAACCGCCACTACT-3ʹ), and reverse primer (5ʹ-ACGCCTAAGCCTGCCTGGTT-3ʹ). All experiments were replicated at least three times with similar results.

### Transgenic DnMSI1 constructs and genetic transformation

To generate the constitutive expression constructs p35S::*DnMSI1* for use in *Arabidopsis thaliana*, the *DnMSI1* coding sequence was PCR-amplified from cDNA using the forward primer: 5ʹ-CTGTCTAGAATGGCCAAGGATGAAGATGAC-3ʹ and reverse primer: 5ʹ-CTGGAGCTCTTAAGAGGCTTTTGAAGGCTCG-3ʹ (the underlined sequences are restriction enzyme sites for *Xba* I and *Sac* I, respectively) and then cloned into the pCAMBIA1300 transformation vector (Takara Biotechnology Co., Ltd., Dalian, China). The resultant 35S*promoter*::DnMSI1 construct was then transformed into *Agrobacterium tumefaciens* strain GV3101, followed by stable transformation into Arabidopsis Col-0, using the floral dip method. Homozygous T3 progenies with *DnMSI1* overexpression (AtOE line) were used for subsequent analysis.

## Results

### Cloning and sequence comparison of DnMSI1

**Figure 1.**
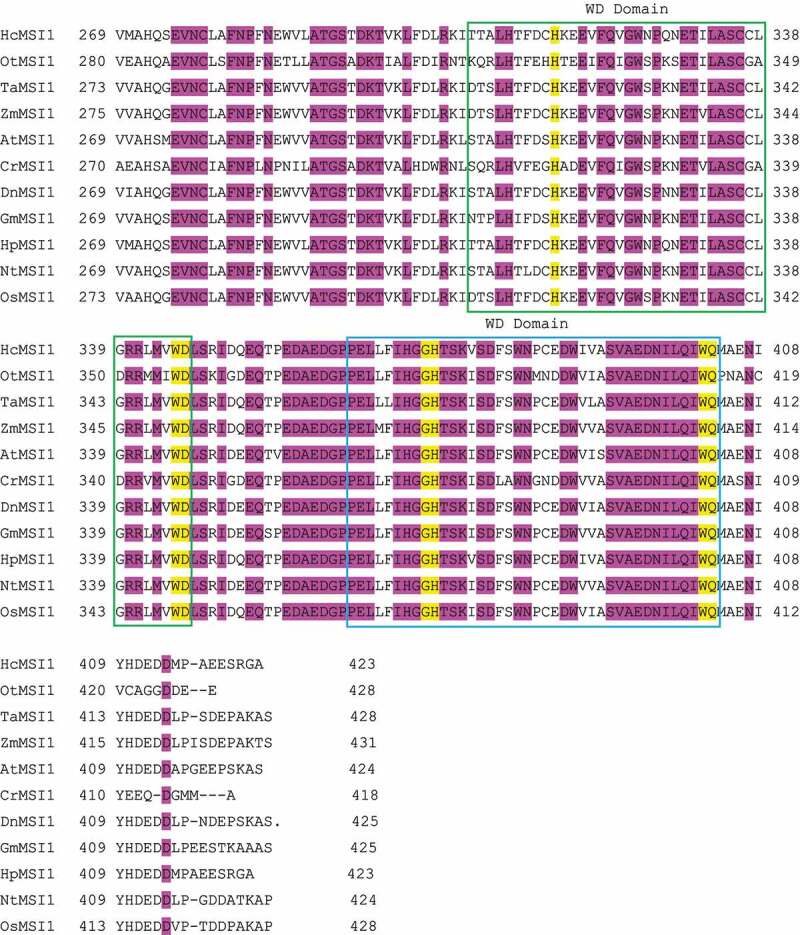
(Continued).

MSI1-like proteins are WD40 repeat proteins that carry one or more WDxR motif(s). The WD domain folds into a β-propeller structure, providing a platform for the protein–protein or protein–DNA interaction and assembly of several proteins.^5^ In this study, the initial 511bp fragment of *DnMSI1* was amplified from *D. nobile* cDNAs, using a primer set of *DnMSI1f1* and *DnMSI1r1*, which were designed with the conserved motifs of *Nicotiana tabacum* MSI1(multicopy suppressor of IRA1) gene (ABY84675.1). Then the 3ʹ and 5ʹ end of *DnMSI1* was isolated with a 3ʹ and 5ʹ rapid amplification of cDNAs ends (RACE). The full-length of *DnMSI1* cDNA was obtained by RT-PCR. Sequence analysis revealed that *DnMSI1* encodes a 424 amino acid polypeptide with theoretical Mr of 48.1kD and *p*I of 4.58, containing four WD40 repeat sequences (Figure 1) according to the WD40 repeat detection method.^15^
**Figure 1.**
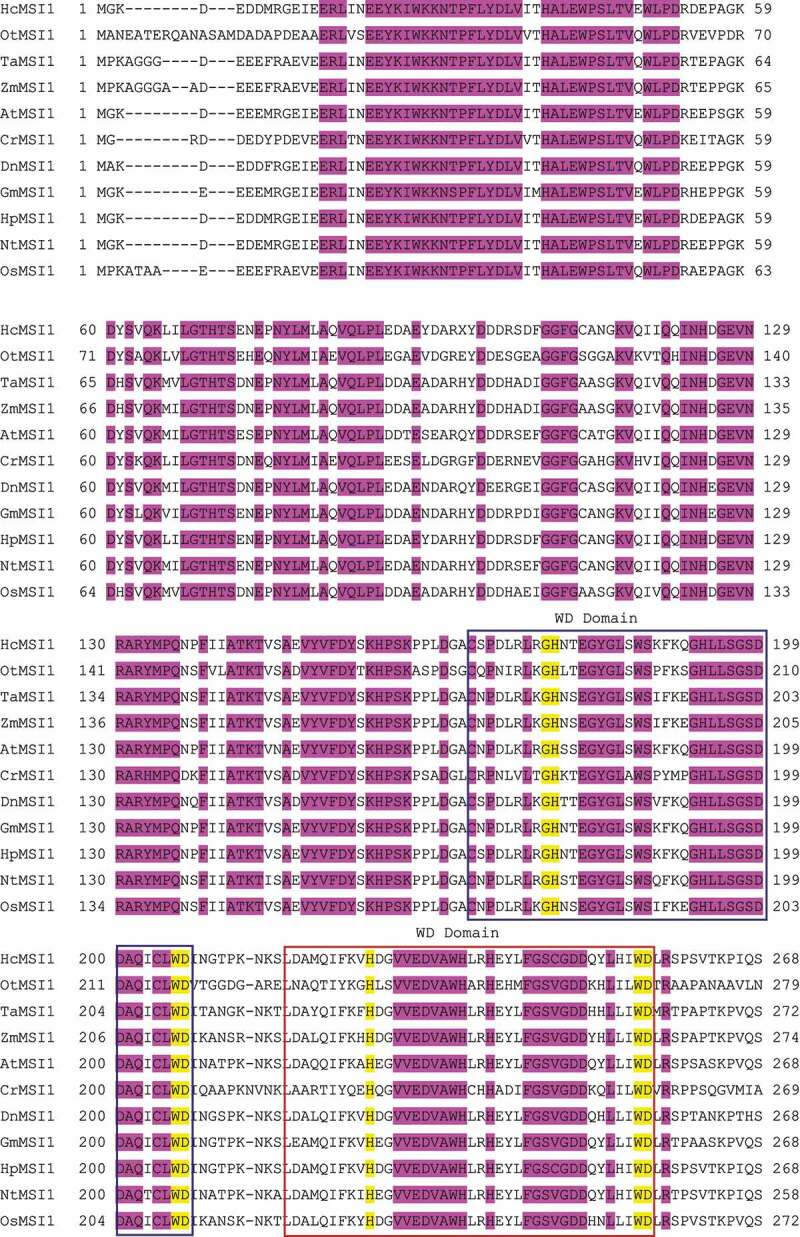
Sequence alignment of DnMSI1 with other closely related MSI1 (Multicopy suppressor of IRA1) proteins. Shaded in purple are amino acids positions identical in all sequences, conserved GH and WD amino acid in WD40 repeat are highlighted in yellow. Squares in different colors indicate the WD Domains. HcMSI1 (*Hieracium caespitosum*, GenBank: ABZ85631.1), OtMSI1 (*Ostreococcus tauri*, XP_003082166.1), TaMSI1 (*Triticum aestivum*, ABB92268.1), AtMSI1 (*Arabidopsis thaliana*, NP_200631.1), CrMSI1 (*Chlamydomonas reinhardtii*, XP_001696907.1), ZmMSI1 (*Zea mays*, NP_001105556.1), GmMSI1 (*Glycine max*, ABW23439.1), HpMSI1 (*Hieracium pilosella*, ABZ85629.1), NtMSI1 (*Nicotiana tabacum*, ABY84675.1), OsMSI1 (*Oryza Sativa*, XP_015632366.1), DnMSI1 (*Dendrobium nobile*).

**Figure 2.**
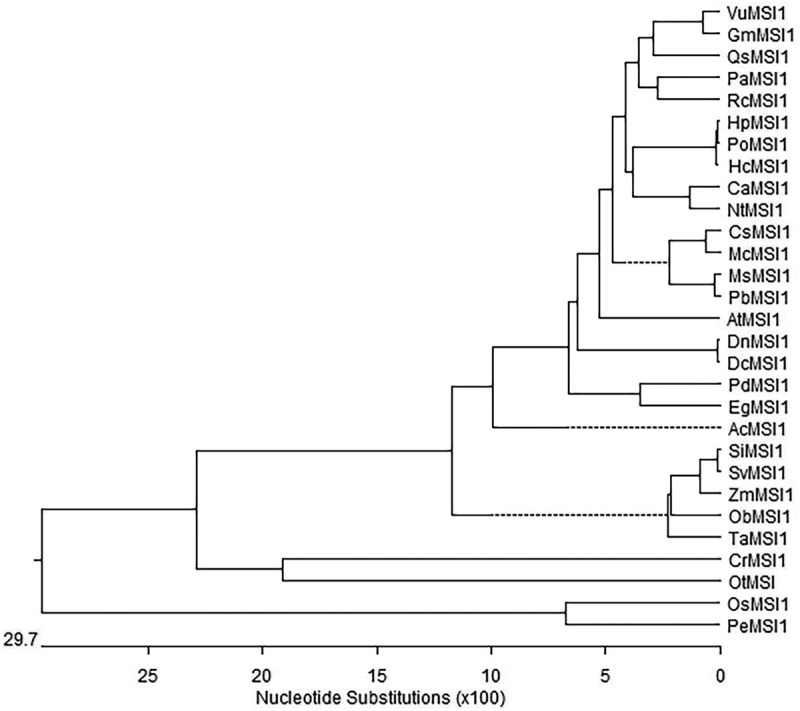
Phylogenetic tree of plant MSI1 proteins from various species. On the scale, the bar 0.1 is equal to 10% sequence divergence. VuMSI1 (*Vigna unguiculata*, XP_017442812), GmMSI1 (*Glycine max*, ABW23439.1), QsMSI1 (*Quercus suber*, XP_023886175.1), PaMSI1 (*Populus alba*, XP_034908184.1), RcMSI1 (*Ricinus communis*, XP_002526518.1), HpMSI1 (*Hieracium pilosella*, ABZ85629.1), PoMSI1 (*Pilosella officinarum*, ABZ85626.1), HcMSI1 (*Hieracium caespitosum*, ABZ85631.1), CaMSI1 (*Coffea arabica*, XP_027113084.1), NtMSI1 (*Nicotiana tabacum*, ABY84675.1), CsMSI1 (*Cucumis sativus*, XP_004133950.1), McMSI1 (*Momordica charantia*, XP_022147047.1), MsMSI1 (*Malus domestica musashi*, XP_028961311.1), PbMSI1 (*Pyrus X bretschneideri*, XP_009376858.1), AtMSI1 (*Arabidopsis thaliana*, NP_200631.1), DnMSI1 (*Dendrobium nobile*), DcMSI1 (*Dendrobium catenatum*, XP_020679078.1), PdMSI1 (*Phoeni X dactylifera*, XP_008794440), EgMSI1 (*Elaeis guineensis*, XP_010923692), AcMSI1 (*Ananas comosus*, XP_020092943.1), SiMSI1 (*Setaria italic*, XP_004982341.1), SvMSI1 (*Setaria viridis*, XP_034575367.1), ZmMSI1 (*Zea mays*, NP_001105556.1), ObMSI1 (*Oryza brachyantha*, XP_006650332.1), TaMSI1 (*Triticum aestivum*, ABB92268.1), CrMSI1 (*Chlamydomonas reinhardtii*, XP_001696907.1), OtMSI1 (*Ostreococcus tauri*, XP_003082166.1), OsMSI1 (*Oryza Sativa*, XP_015632366.1), PeMSI1 (*Phalaenopsis equestris*, XP_020572556.1).

The deduced protein sequence shows high similarity with MSI1 from the other plants with 97, 89, 86, 82, and 81% identity to *Dendrobium catenatum* DcMSI1, *Arabidopsis thaliana* AtMSI1, *Nicotiana tomentosiformis* NtMSI1, *Zea mays* ZmMSI1, and *Oryza sativa* OsMSI1, respectively. To investigate the phylogenetic relationship of the MSI1 proteins, a phylogenetic tree was constructed for several other MSI1 orthologs. The results revealed that DnMSI1 is closely related to DcMSI1 of *Dendrobium catenatum* (Figure 2).

### Expression profile of DnMSI1 transcript in D.nobile Lindl

**Figure 3.**
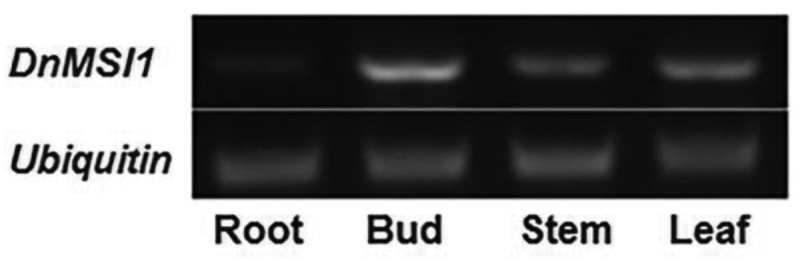
Expression profiles of *DnMSI1*. Semi-quantitative RT-PCR analysis of the expression level of *DnMSI1* in different tissues under normal conditions. The *D. nobile* ubiquitin gene was amplified as an internal control.

**Figure 4.**
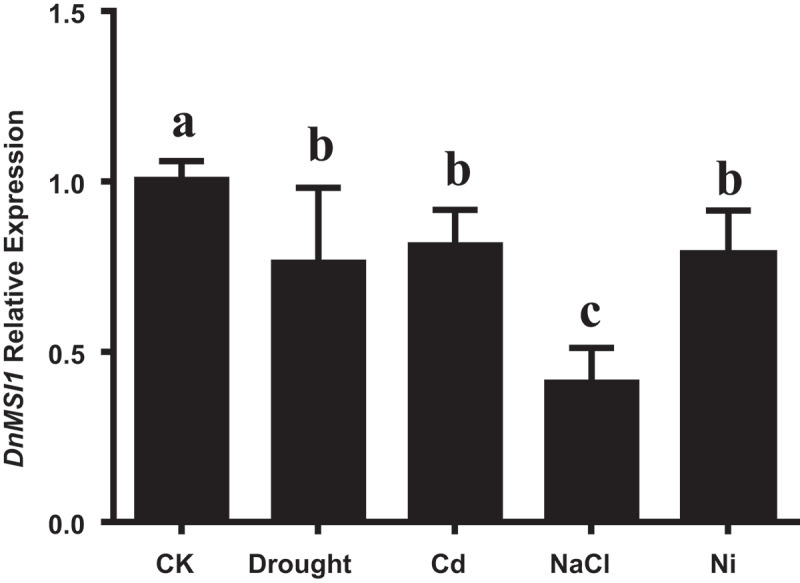
The expression level of *DnMSI1* after drought, Cd, Ni, and NaCl treatment. Distilled water as a negative control. The *D. nobile* ubiquitin gene was amplified as an internal control for normalization of *DnMSI1* mRNA levels. Data are means ± SD (n = 3). Different letters above the columns indicate statistically significant differences at P < .05 by Tukey’s test.

Semi-quantitative RT-PCR analysis showed that the transcript of *DnMSI1* was detected in buds, leaves, and stems, but hardly detected in roots. *DnMSI1* expression is more robust in buds than in stems and leaves (Figure 3). To determine whether abiotic stress regulated *DnMSI1* transcription, quantitative RT-PCR was performed. Compared to the control condition, the levels of *DnMSI1* expression in buds were decreased 25, 19, 22, and 59% by drought, Cd, Ni, and salt stress, respectively (Figure 4). The results suggested that DnMSI1 is negatively regulated by abiotic stresses, including drought, salt, and heavy metal stresses.

**Figure 5.**
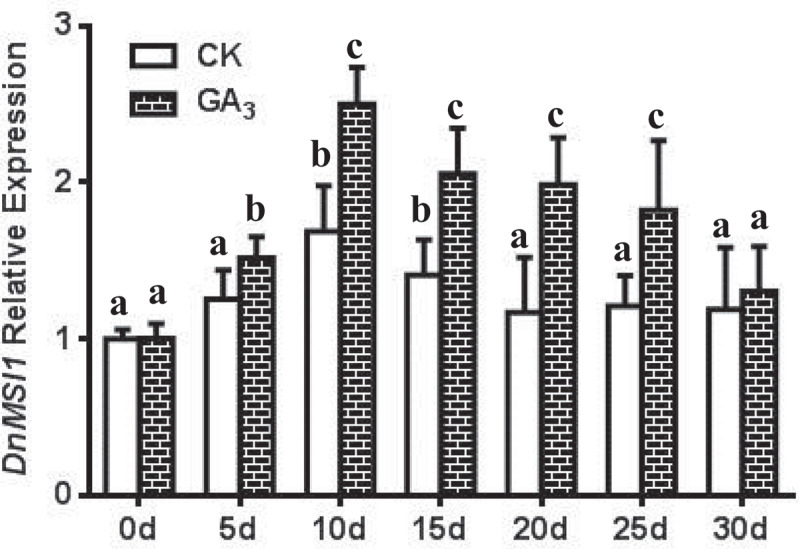
Time-dependent expression pattern of *DnMSI1* in buds by GA_3_, distilled water as a negative control. *D. nobile* ubiquitin gene was amplified as an internal control for normalization of *DnMSI1* mRNA levels. Data are means ± SD (n = 3). Different letters above the columns indicate statistically significant differences at P < .05 by Tukey’s test.

**Figure 6.**
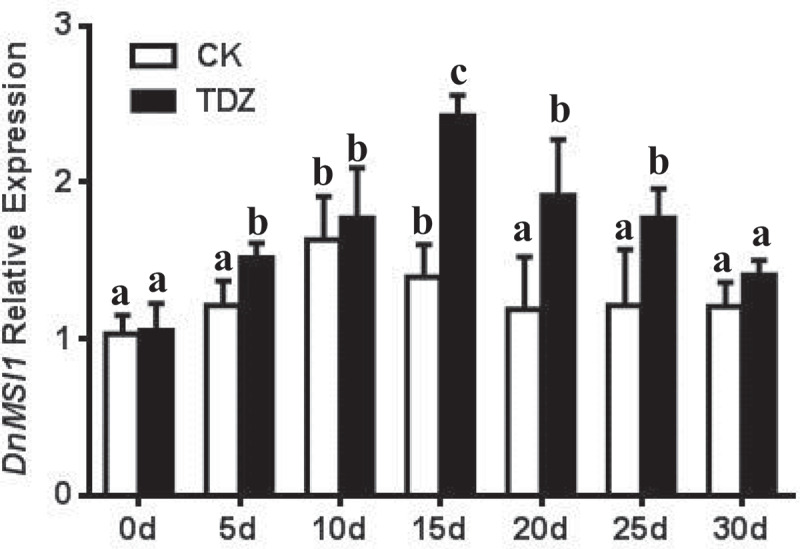
Time-dependent expression pattern of *DnMSI1* in TDZ-treated buds. Distilled water as a negative control. *D. nobile* ubiquitin gene was amplified as an internal control for normalization of *DnMSI1* mRNA levels. Data are means ± SD (n = 3). Different letters above the columns indicate statistically significant differences at P < .05 by Tukey’s test.

**Figure 7.**
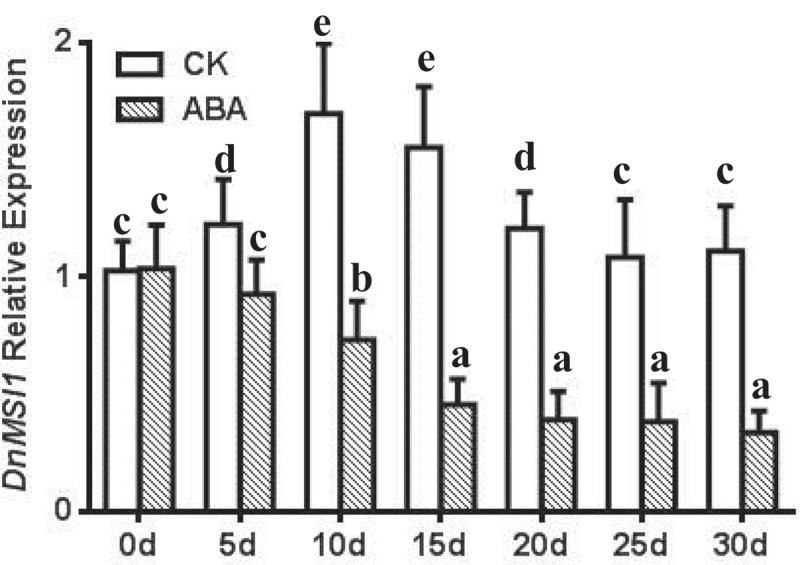
Time-dependent expression pattern of *DnMSI1* in buds by ABA, distilled water as a negative control. *D. nobile* ubiquitin gene was amplified as an internal control for normalization of *DnMSI1* mRNA levels. Data are means ± SD (n = 3). Different letters above the columns indicate statistically significant differences at P < .05 by Tukey’s test.

**Figure 8.**
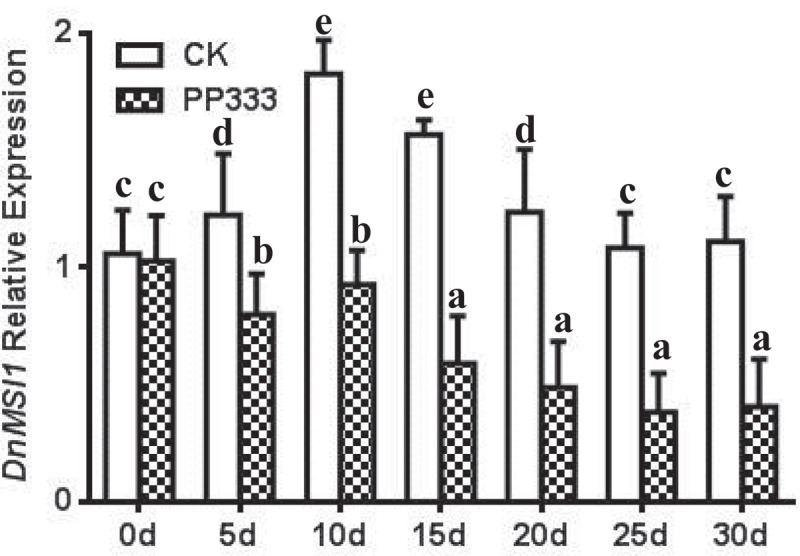
Time-dependent expression pattern of *DnMSI1* in buds by PP333, distilled water as a negative control. *D. nobile* ubiquitin gene was amplified as an internal control for normalization of *DnMSI1* mRNA levels. Data are means ± SD (n = 3). Different letters above the columns indicate statistically significant differences at P < .05 by Tukey’s test.

Interactions of DnMSI1 protein with plant hormones and regulators and their roles in regulating *D. nobile* development were further investigated. Four types of hormones or plant regulators, i.e., GA3, thidiazuron (TDZ), ABA, and PP333, were selected, and *DnMSI1* expression in response to the hormone and growth regulator treatment was analyzed at different developmental stages. The level of *DnMSI1* transcripts was up-regulated post GA_3_ and TDZ treatment and peaked at 10d or 15d, respectively (Figures 5, 6). In contrast, ABA and PP333 treatment down-regulated *DnMSI1* expression after 5-d treatment compared to normal condition (Figures 7, 8). These results demonstrated that DnMSI1 responds differently to plant growth regulators or hormones.

### Constitutive DnMSI1 expression in A. thaliana decreases tolerance to salinity

**Figure 9.**
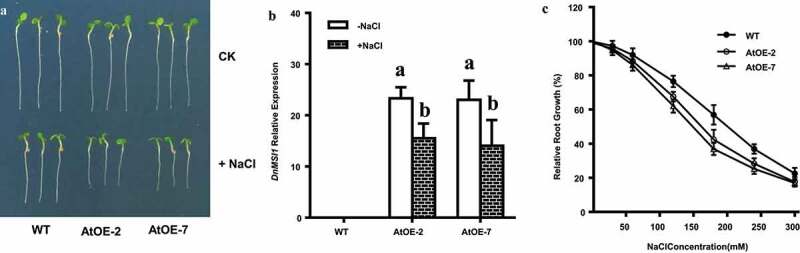
*DnMSI1*over-expression Arabidopsis lines are more sensitive to NaCl stress. (a) NaCl sensitivity of WT and two AtOE lines. Seedlings were treated with 0 (CK) or 100 mM NaCl for 3 d. (b) *DnMSI1* relative expression in wild type Arabidopsis(WT) and *DnMSI1*over-expression transgenic lines (AtOE-2 and AtOE-7). Data are means ± SD (n = 3). Different letters above the columns indicate statistically significant differences at P < .05 by Tukey’s test. (c) Relative root growth (RRG%) of individual lines. Data are means ± SD (n = 10).

To explore the effect of *DnMSI1* expression on plant responses to salinity stress, *DnMSI1* was constitutively expressed in *Arabidopsis thaliana* (AtOE lines). Quantitative RT-PCR analysis showed that the *DnMSI1* transcript levels are significantly higher in AtOE lines than that in wild-type (WT) *Arabidopsis*, when calibrated with the internal reference gene (Figure 9b). In addition, the *DnMSI1* expression is decreased by NaCl in AtOE lines, but its transcript levels are still much higher than that in WT *Arabidopsis* (Figure 9b).

The responses to different NaCl concentrations were further compared between two independent *DnMSI1* AtOE lines and the WT control line. Under the standard condition without stresses (CK), no phenotypic differences were observed among the lines (Figure 9a), which indicated that over-expression of *DnMSI1* in Arabidopsis has no obvious effect on the growth of Arabidopsis seedlings at standard condition. However, the two independent *DnMSI1*-overexpression Arabidopsis (AtOE) lines were comparably more sensitive to NaCl stress than the WT (Figure 9a). At 180 mM NaCl, the root growth of WT was inhibited by 43%, whereas the AtOE lines by 58 and 65%, respectively (Figure 9c). These findings indicated that the *DnMSI1* over-expression transgene notably decreases the Arabidopsis plants’ tolerance to NaCl stress, and DnMSI1 plays a negative regulatory role in plant response to salinity stress.

## Discussion

MSI1-like proteins play essential roles in plant development and various aspects of chromatin assembly. MSI1 functions are conserved across plant species. Altered levels of *MSI1* expression result in pleiotropic phenotypes, reflecting the complexity of MSI1 protein functions.^10^ In this study, we identified an MSI1 homolog in orchid *D.nobile* Lindl, and the DnMSI1 protein has four WD40 characteristic domains (Figure 1) and close relation to DcMSI1 of *Dendrobium catenatum* (Figure 2).

Like plant hormones or plant growth regulators, MSI1 is required for regulating plant development throughout plant life.^1,6,7,11^ GAs play pivotal roles in many developmental processes in plants, including pollen maturation, seed germination, leaf expansion, stem elongation, flowering induction, and trichome development.^16,17^ *DnMSI1* expression was enhanced by plant hormone Gibberellin (GA_3_), reaching the maximum level 10 days after the treatment. Then, *DnMSI1* expression levels in the buds gradually decreased but were still higher than the control condition 30 days after the treatment. Moreover, *DnMSI1* expression was promoted by TDZ (thidiazuron), a cytokinin-like plant regulator. However, the expression peak was delayed compared with that of GA_3_-treatment. These results suggest that TDZ regulates *DnMSI1* expression differently from GA.

By contrast, the expression of *DnMSI1* was decreased after applying the plant growth inhibitor, Paclobutrazol (PP333). It has been well documented that PP333 can inhibit the biosynthesis of plant gibberellin (GA) to delays plant growth and development.^16^ So, GA could up-regulate the expression of *DnMSI1* directly in *D.nobile* Lindl, and PP333 down-regulate *DnMSI1* expression through inhibition of GA biosynthesis.

As a plant growth retardant, Abscisic acid (ABA) regulates diverse plant growth and development processes under stress conditions. It plays a crucial role in abiotic stress tolerance by mediating genetic and epigenetic processes in plant stress responses.^18^ In this study, we found that *DnMSI1* expression was suppressed by exogenously applied ABA and by salt, drought, and heavy metal (Cd, Ni) stresses. The Arabidopsis MSI1 suppresses the expression of ABA-responsive genes, specifically salt and osmotic stress-related genes, and plays negative roles in regulating drought stress responses.^13^ MSI1 is involved in chromatin dynamics and inheritance of epigenetic states during mitosis.^19^ When over-expressing DnMSI1 in Arabidopsis, the transgenic plant lines exhibited obvious sensitivities to NaCl stress. These results suggest that an epigenetic response mechanism could be triggered through ABA-regulated and MSI1-mediated chromatin assembly and dynamics in response to abiotic stresses, leading to negative regulation of salinity stress responses in the plant.

## Data Availability

The data supporting this study’s findings are available from the corresponding author, Y.W., upon reasonable request.
